# Quality of Life, Sleep Quality, Depression, Anxiety, Stress, Eating Habits, and Social Bounds in Nurses during the Coronavirus Disease 2019 Pandemic in Qatar (The PROTECTOR Study): A Cross-Sectional, Comparative Study

**DOI:** 10.3390/jpm11090918

**Published:** 2021-09-15

**Authors:** Abdulqadir J. Nashwan, Ralph C. Villar, Ahmad R. Al-Qudimat, Nisha Kader, Majid Alabdulla, Ahmad A. Abujaber, Mahmood M. Al-Jabry, Michel Harkous, Anite Philip, Raed Ali, Prem Chandra, Mohamed A. Yassin, Mujahed Shraim, Kalpana Singh

**Affiliations:** 1 Department of Nursing, Hazm Mebaireek General Hospital (HMGH), Hamad Medical Corporation (HMC), Doha 3050, Qatar; RVillar@hamad.qa (R.C.V.); aabujaber@hamad.qa (A.A.A.); MAlJabry@hamad.qa (M.M.A.-J.); 2 Faculty of Nursing, University of Calgary in Qatar, Doha 23133, Qatar; 3 Surgical Research Section, Department of Surgery, Hamad Medical Corporation (HMC), Doha 3050, Qatar; AAlQudimat@hamad.qa; 4 Mental Health Services (MHS), Hamad Medical Corporation (HMC), Doha 3050, Qatar; nishakeem@gmail.com (N.K.); malabdulla3@hamad.qa (M.A.); 5 College of Medicine, Qatar University, Doha 2713, Qatar; 6 Department of Nursing, National Center for Cancer Care and Research (NCCCR), Hamad Medical Corporation (HMC), Doha 3050, Qatar; MHarkous@hamad.qa (M.H.); APhilip8@hamad.qa (A.P.); 7 Department of Nursing, Heart Hospital (HH), Hamad Medical Corporation (HMC), Doha 3050, Qatar; RAli12@hamad.qa; 8 Medical Research Center, Academic Health System (AHS), Hamad Medical Corporation (HMC), Doha 3050, Qatar; PChandra@hamad.qa; 9 National Center for Cancer Care and Research (NCCCR), Hamad Medical Corporation (HMC), Doha 3050, Qatar; Yassin@hamad.qa; 10 Department of Public Health, College of Health Sciences, QU Health, Qatar University, Doha 2713, Qatar; mshraim@qu.edu.qa; 11 Nursing & Midwifery Research Department, Hamad Medical Corporation (HMC), Doha 3050, Qatar; KSingh1@hamad.qa

**Keywords:** nurses, Coronavirus Disease 2019 (COVID-19), quality of life, eating habits, social support

## Abstract

There have been numerous concerns regarding the physical and mental health of nurses during the COVID-19 pandemic. Stress, sleep deprivation, anxiety, and depression potentiated nurses’ vulnerability to poor eating habits. Aims and Objectives: The purpose of this study was to explore the differences between nurses’ characteristics with COVID-19 facility designation, and sleep quality, depression, anxiety, stress, eating habits, social bonds, and quality of life. Design: A cross-sectional, comparative study. Methods: An online survey was sent using the corporation’s email to nurses working in three hospitals in Qatar from September to December 2020. One of them is a designated COVID-19 facility. The sleep quality, depression, eating habits, social bonds, and quality of life were measured using The Insomnia Severity Index (ISI), Depression Anxiety and Stress Scale 21 (DASS-21), Emotional Eater Questionnaire (EEQ), Oslo Social Support Scale (OSSS-3), and the World Health Organization Quality of Life (WHOQOL-BREF), respectively. Results: A total of 200 nurses participated in the study (RR: 13.3%). No statistically significant association was found between designated facility (COVID-19 vs. not COVID-19) or nurses’ characteristics and ISI categories (OR 1.15; 95% CI 0.54, 2.44). Nurses working in COVID-19 facilities had increased odds of having higher EEQ categories by 2.62 times (95% CI 1.18, 5.83). Similarly, no statistically significant associations were found between any of the nurses’ characteristics and OSSS-3 categories. On the other hand, no statistically significant associations were found between any of the nurses’ characteristics and QOL domains except for the gender and social relationships’ domain. Conclusion: Overall, the quality of life of nurses in Qatar is on a positive level whether they are assigned to a COVID-19 facility or not. Although no significant difference was found with regard to the sleep quality, stress, anxiety, depression, and eating habits between nurses in a COVID-19 facility and in a non-COVID-19 facility, special interventions to diminish stressors need to be implemented and maintained.

## 1. Introduction

Qatar is one of the wealthiest, most multicultural countries in the world, with a rapidly growing population representing over 80 nationalities. In Qatar, nurses and midwives per 1000 people were reported at 7.2628 in 2018, according to the World Bank collection of development indicators. The majority (>90%) of the nurses are non-Qatari and working at Hamad Medical Corporation (HMC) (>12,000), the largest healthcare facility in Qatar [1,2].

The sudden surge in Coronavirus Disease 2019 (COVID-19) cases brought significant demands for nurses worldwide. Nurses were immediately assigned to care for patients with the coronavirus, which was not completely understood. Nurses had to quickly adjust to new clinical pathways and policies, master new nursing skills, and report to substandard working environments with less or zero time for training and orientation [3]. Furthermore, they had to wear personal protective equipment (PPE) for extended hours, which caused additional stress, pressure injuries, and discomfort [4,5,6,7,8,9]. There have been numerous reports of physical and mental health problems among nurses during the coronavirus pandemic brought by insomnia, stress, anxiety, and depression [8,10,11]. A recent meta-analysis found a high prevalence of anxiety, depression, and sleep disturbance among nurses during the COVID-19 pandemic compared to the general public (37%, 35%, 43%, respectively), which is higher than that of previous epidemics such as MERS and SARS. This could be because of the high contagiousness and fatality of the virus causing millions of deaths worldwide [12,13]. Globally, the International Council of Nurses (ICN) reported around 2262 COVID-19 related deaths and more than 1.6 million COVID-19 infections among nurses [14]. Furthermore, a majority of nurses witnessed patients suffering and dying alone because family visits were prohibited to prevent further spread of the infection. The therapeutic relationship and the specialized type of care evolved an emotional impact for nurses caring for prolonged suffering patients with COVID-19 in intensive and emergency care units, which can contribute to depression among nurses [15].

Change in eating habits was another common coping mechanism for nurses; this could be in the form of consuming high energy-dense foods that have a significant correlation with weight gain [16,17,18,19].

Social support has become an important factor to cope with the challenges of COVID-19. Research evidence suggests that social interactions with family, friends, neighbors, and other health care professionals reduce anxiety and stress, and improve self-efficacy among nurses [10]. However, most healthcare workers in Qatar are expatriates working away from their family and friends. In addition, with the implementation of strict rules on quarantine and lockdowns, it could be more challenging to find support from significant persons. For example, despite the successful control of coronavirus transmission in New Zealand, strict lockdowns were associated with significant negative impact on mental health in the general population [20].

Overall, this increases the probability of low quality of life among nurses [21,22]. Quality of life is a broad-range concept that can be affected by a person’s physical and psychological health, level of independence, social relationships, and relationships to salient features of their environment [23]. The World Health Organization (WHO) defines quality of life as the individual’s perception of their position in life in the context of the culture and value systems in which they live and in relation to their goals, expectations, standards, and concerns [24].

Several longitudinal studies confirmed the impact of COVID-19 on the physical, mental, spiritual, and social aspects of nurses’ lives [25,26,27,28,29].

Nurses in Qatar have been working tirelessly since the beginning of the pandemic. As part of the national response to COVID-19, most nurses in Qatar were assigned to work in COVID-19 facilities. The COVID-19 facilities were fully functioning hospitals designated by the Ministry of Public Health in Qatar (MoPH) to treat moderate- to- severe cases of COVID-19. Little is known about the impact of COVID-19 on sleep quality, depression, eating habits, social bonding, and quality of life in nurses in Qatar, and whether working in a COVID-19 facility has an impact on these factors or not. Specifically, the study aims to explore the differences between nurses’ characteristics with COVID-19 facility designation and sleep quality, depression, anxiety, stress, eating habits, social bonds, and quality of life. The findings of this study may lead to a better understanding of the effects of the COVID-19 crisis on frontline nurses and thereby serve as a basis to improve work aspects.

## 2. Materials and Methods

### 2.1. Data Collection and Settings

An online survey was sent using the corporation’s official email to nurses working in three specified hospitals from September to December 2020 (between the 1st and 2nd wave in Qatar where the number of cases started to decline before the 2nd wave). According to the World Health Organization, there are 142,903 accumulated confirmed cases and 244 deaths in Qatar (updated 29 December 2020) [30]. One of the hospitals was Hazm Mebaireek General Hospital (HMGH), a designated COVID-19 facility in Qatar. HMGH has a 118-bed capacity and more than 500 nurses to treat moderate-to-severe cases of COVID-19. It is a fully functioning tertiary hospital that was remodeled to receive hundreds of critically ill patients. The other two hospitals were no-COVID-19 facilities: The Heart Hospital (HH) (a specialist hospital in Qatar for cardiology and cardiothoracic surgery patients made up of a state-of-the-art 20-bed coronary care unit, an equally impressive 12-bed cardiothoracic intensive care unit (ICU), a 24-bed surgical high-dependency unit (HDU), and a 60-bed ward with more than 200 nurses), and the National Center for Cancer Care & Research (NCCCR) (the premier cancer hospital in Qatar, which is part of HMC and looks after cancer patients who require ongoing treatments such as chemotherapy and radiotherapy with a 50-bed capacity and more than 100 nurses).

### 2.2. Instruments

The sleep quality, depression, eating habits, social support, and quality of life were measured using the Insomnia Severity Index (ISI), Depression Anxiety and Stress Scale 21 (DASS-21), Emotional Eater Questionnaire (EEQ), Oslo Social Support Scale 3 (OSSS-3), and the World Health Organization Quality of Life (WHOQOL-BREF), respectively. The ISI is a valid and reliable (Cronbach alpha = 0.84; *r* = 0.65–0.84 for discriminative capacity) 7-item self-reporting tool to measure one’s perception of his or her insomnia [31].

The DASS-21 is a 21-item self-reporting scale designed to measure the emotional states of depression, anxiety, and stress [32]. Each of the three emotional states contains 7 questions divided into subscales with similar content. Multiple studies have done psychometric assessments on DASS-21 and in different languages, and concluded that DASS-21 has a good-to-excellent validity and consistency [33,34,35,36,37,38]. In one study, the reliability of DASS-21 showed excellent Cronbach’s alpha values of 0.81, 0.89 and 0.78 for the subscales of depressive, anxiety, and stress, respectively [39].

The EEQ is a 10-item questionnaire that assesses the extent of how emotions affect eating behavior [40]. Each item has four possible answers (never = 0, sometimes = 1, generally = 2, and always = 3). Total scores are then classified into four groups: non-emotional eater (0–5), low emotional eater (6–10), emotional eater (11–20), and very emotional eater (21–30). To analyze factor structure and psychometric properties of Emotional Eater Questionnaire (EEQ), the authors recruited 295 participants, and Exploratory Factor Analysis (EFA) was carried out by using the Unweight Least Squares (ULS) method [41]. The parallel analysis and goodness of fit statistics showed that unifactorial structure of seven items was the most appropriate and accounted for 57% of the variance. Internal consistency was good (α = 0.753), as well as convergent validity (*r* = 0.317; *p* < 0.001). Concurrent validity was significant for three of the five criteria (*r* = −0.224; *p* < 0.001 and *r* = −0.259; *p* < 0.001).

The OSSS-3 consists of three items [42], which assess the number of close confidants, the sense of concern from other people, and the relationship with neighbors, with a focus on the accessibility of practical help [43]. In Kocalevant’s (2018) assessment of OSSS-3, the internal consistency was acceptable with *α* = 0.640. In addition, an EFA was also conducted to determine the construct validity of the OSSS-3 based on the correlative associations between its items [43].

The WHOQOL-BREF is a shortened version (26-item) of the WHOQOL-100, which assesses the quality of life in epidemiological and clinical studies [44,45]. Cheung (2017) recruited 1316 participants to test the reliability and validity of WHOQOL-BREF in three different languages. The Tucker–Lewis Index (TLI) was 0.919, 0.913, and 0.909 for the English, Chinese and Malay versions, respectively. Standardized root mean square residual (SRMR) was 0.067, 0.074, and 0.094, respectively. Cronbach’s alpha exceeded 0.7, and ICC exceeded 0.4 for all domains in all language versions, making WHOQOL-BREF a valid and reliable tool [45].

Overall, the tools used in this research study had good-to-excellent validity and reliability to assess the different variables.

### 2.3. Inclusion Criteria

The survey link was sent to approximately 1500 nurses via the corporation’s nursing e-mail group. We assigned a two-month period for completing the survey. During this period, we counted how many persons clicked the link.

The survey includes the participants’ demographics: gender, age, marital status, living status, hospital, unit, job title, and COVID-19 facility designation (yes, no).

Only nurses who were working in the selected hospitals were included and those who were working in HMC for the past year and were willing to participate.

### 2.4. Statistical Analysis

Numbers with percentages were used to summarize the characteristics of nurses. Multivariable ordinal regression was used to assess the relationship between nurses’ characteristics with ISI, EEQ, and OSSS-3 categories. Multivariable linear regression was used to assess the relationship between nurses’ characteristics with QOL, depression, anxiety, and stress scores. Values of *p* < 0.05 were considered statistically significant (two-sided tests). All data were analyzed using STATA 15.0 statistical software.

## 3. Results

A total of 200 nurses participated in the study (response rate 13.3%) (Table 1). About 74.5% of nurses were working in COVID-19 facilities. Around 19.0%, 65.5%, and 15.5% of nurses were aged 20–30, 31–40, and above 40 years, respectively. About 59.5% and 80.0% of nurses were males and married, respectively. Around 61.0%, 21.5%, and 17.5% of nurses had 1–3, 4–10, and more than 10 years of nursing experience in Qatar, respectively.

### 3.1. Adjusted Associations between Characteristics of Nurses and ISI, EEQ, and OSSS-3 Categories

As shown in Table 2, no statistically significant association was found between designated facility (COVID-19 vs. not COVID-19) and ISI categories (OR 1.15; 95% CI 0.54, 2.44; *p* = 0.726). Similarly, no statistically significant associations were found between other nurses’ characteristics (age, gender, marital status, and nursing experience) and ISI categories.

Nurses working in COVID-19 facilities had increased odds of having higher EEQ categories by 2.62 times (95% CI 1.18, 5.83; *p* = 0.018) than nurses working in non-COVID-19 facilities. In addition, male and married nurses had lower odds of having higher EEQ categories by 0.37 (95% CI 0.19, 0.72; *p* = 0.003) and 0.36 (0.17, 0.74; *p* = 0.006) than female and single nurses, respectively.

No other statistically significant associations were observed between nurses’ characteristics (age and nursing experience) with EEQ categories. Similarly, no statistically significant associations were found between any of the nurses’ characteristics and OSSS-3 categories (Table 2).

### 3.2. Adjusted Associations between Characteristics of Nurses and QOL Domains, Depression, Anxiety, and Stress Scores

As shown in Table 3, no statistically significant associations were found between any of the nurses’ characteristics and QOL domains except for the gender and social relationships’ domain. Married nurses had a higher score of QOL than single nurses in the social relationships’ domain by 10.8 (95% CI 3.4, 18.2; *p* = 0.007). Most nurses had normal or mild symptoms severity of depression (96%), anxiety (86%), and stress (98%). Therefore, the relationships between characteristics of nurses and DASS-21 scores were assessed using multiple linear regression instead of ordinal regression. As shown in Table 3, no statistically significant associations were found between any of the nurses’ characteristics with symptoms severity scores of depression, anxiety, and stress.

## 4. Discussion

The aim of the study was to research the differences between nurses’ characteristics with COVID-19 facility designation (yes, no) and sleep quality, depression, anxiety, stress, eating habits, social bonds, and quality of life. While multiple studies have been published on sleep quality and depression, to our knowledge this is the first study to investigate eating habits, social support, and quality of life among nurses during the COVID-19 pandemic.

In this study, there were no significant differences in sleep quality, stress, anxiety, depression, and perceived social support between nurses working in COVID-19 and non-COVID-19 facilities in Qatar, and this might be explained by several possible factors such as the level of support provided by the nursing management or the “timing”, as the survey was disseminated after the first wave where the nurses started to adapt to the “new normal”, especially in the absence of a proper pre-pandemic control group. Interestingly, most nurses in COVID-19 and non-COVID-19 facilities had normal or mild depression, anxiety, and stress symptoms severity. This is in contrast with previous studies performed in Korea and China showing significant differences in sleep quality, stress, anxiety, and depression levels between health care workers who were exposed to COVID-19 and those were not [25,26]. A possible explanation could be related to several factors. Firstly, nurses in Qatar perceived the same level of administrative support in both COVID-19 and non-COVID-19 facilities, such as the provision of PPE, medical supplies, and equipment [25,46]. Secondly, there were noticeably fewer confirmed COVID-19 death cases in Qatar compared to other countries with high rates of anxiety and depression among nurses [12,47,48,49,50]. Alnazly et al. reported a positive relation in the level of psychological discomfort (stress and anxiety) among nurses who were working in COVID-19 facilities and were over the age of 40 [51]. The increased degree of anxiety among older workers could be further explained by the fear of respiratory distress infection from COVID-19 [51]. This is in contrast with our study, as the majority of participants were younger nurses and below the age of 40. Furthermore, Alnazly et al. and Labrague et al. found that COVID-19 anxiety has also been tackled by social and organizational support [51,52]. These findings support the current study by showing that most nurses in COVID-19 and non-COVID-19 facilities had normal or mild depression, anxiety, and/or stress symptoms. Our result could be explained as nurses in Qatar perceived the same level of administrative support in both COVID-19 and non-COVID-19 facilities.

Mota and co-workers found that eating habits have a negative impact on Brazilian healthcare professionals, in which 78.5% of respondents changed their diet, 24.5% reported an increase in carbohydrate intake, and 27% reported an increase in the consumption of alcoholic beverages [53]. The results support the findings of the current study, as we found that nurses working at COVID-19 facilities in Qatar had increased odds of having higher EEQ categories than nurses working in non-COVID-19 facilities. In addition, male and married nurses had lower odds of having higher EEQ categories than female and single nurses, respectively. This could be due to the high workload and high risk of exposure in a COVID-19 facility [8,10,12,16,17,46,54,55]. However, baseline data are required to further investigate and correlate with COVID-19, especially when no associated significant increase in the levels of stress and anxiety were found.

Xiao et al. and Yu et al. reported that positive coping strategies and increased social support were both found to be associated with lower levels of anxiety and stress, better sleep quality, and lower levels of psychological distress among nurses [10,56]. These findings were also parallel with our study showing that married nurses had a higher score of QOL in the social relationships’ domain than single nurses. In addition, there are numerous factors that are thought to affect QOL in the social relationship domain among single nurses in Qatar. One reason could be that most workers in Qatar are expatriates with a high likelihood that their families and friends are in their home country. Furthermore, the physical restrictions on social distancing under government policies can exacerbate feelings of loneliness [33].

The findings of this study could have several suggestions for interventions aimed at improving and maintaining nurses’ psychological well-being during pandemics. Institutions should offer good strategic plans and counseling support services, as well as training materials and online workshops to help nurses overcome any psychological issues [57]. Continuing evaluation of the psychological interventions during a pandemic to alleviate depression, stress, and anxiety should be provided for nurses regardless of their work assignment. Furthermore, they should enhance the working conditions of nurses by resource allocation and increased manpower. It is also recommended that they encourage their families and friends to boost the social support provided to nurses during high-risk periods such as pandemics, to enhance QOL in social relationships and minimize or prevent the adverse effects of stress [9,28,29,30,31,32].

### Limitations

The first limitation of this study was the scope. Most of the participants who responded were from a single COVID-19 facility; the respondents from a non-COVID-facility were only 36% (51 nurses), which could lead to bias and which limits generalization of the findings. Second is the possibility of selection bias. Although we sent the invitation to over 1500 nurses via the corporate e-mail, the non-respondents were either too stressed to complete the survey or not interested at all in participating (the response rate was very low, 13.3%). Third, the survey was carried out months after the peak of COVID-19; the response may have been different if the study was done during the critical stage of the pandemic. Fourth, the utilized tools were not validated previously in Qatar, which might jeopardize the generatability as well, even though the vast majority of the nursing workforce in Qatar are expatriates.

## 5. Conclusions

Overall, the quality of life of nurses in Qatar is on a positive level, whether they are assigned in a COVID-19 facility or not. Although we did not find any significant difference in the sleep quality, stress, anxiety, depression, and perceived social support between nurses in COVID-19 and non-COVID-19 facilities, nursing leaders should keep protecting the physical and mental health of nurses to take a more active role in maintaining and improving their health during tough times such as COVID-19. Special environmental and individual-based intervention programs to diminish stressors and to encourage social bounding need to be implemented and maintained.

## Figures and Tables

**Table 1:** Characteristics of participants.

	Number (%)
Gender	
Female	81 (40.5)
Male	119 (59.5)
Age	
20–30	38 (19.0)
31–40	131 (65.5)
>40	31 (15.5)
Marital Status	
Married	160 (80.0)
Single	40 (20.0)
Years of Experience in Qatar	
1–3 years	122 (61.0)
4–10 years	43 (21.5)
>10 years	35 (17.5)
Facility	
COVID-19	149 (74.5)
Non-COVID-19	51 (25.5)

**Table 2:** Adjusted associations between characteristics of nurses and ISI, EEQ, and OSSS-3 categories.

	ISI Category						EEQ Category						OSSS-3 Category				
	No Clinically Significant N (%)	Subthreshold N (%)	Moderate Severity N (%)	Severe N (%)	OR (95% CI)	*p*	Non-Emotional Eater N (%)	Low Emotional Eater N (%)	Emotional Eater N (%)	Very Emotional Eater N (%)	OR (95% CI)	*p*	Poor Social Support N (%)	Moderate Social Support N (%)	Strong Social Support N (%)	OR (95% CI)	*p*
Age																	
20–30	6 (15.8)	23 (60.5)	8 (21.1)	1 (2.6)	Ref		13 (34.2)	8 (21.1)	15 (39.5)	2 (5.3)	Ref		11 (29.0)	18 (47.4)	9 (23.7)	Ref	
31–40	31 (23.7)	62 (47.3)	31 (23.7)	7 (5.3)	0.84 (0.39, 1.78)	0.642	47 (35.9)	44 (33.6)	35 (26.7)	5 (3.8)	0.81 (0.37, 1.76)	0.591	57 (43.5)	46 (35.1)	28 (21.4)	0.64 (0.29, 1.38)	0.251
>40	11 (35.5)	14 (45.2)	6 (19.4)	0 (0.0)	0.61 (0.16, 2.34)	0.468	12 (38.7)	13 (41.9)	5 (16.1)	1 (3.2)	0.54 (0.13, 2.13)	0.376	9 (29.0)	19 (61.3)	3 (9.7)	0.92 (0.25, 3.47)	0.905
Gender																	
Female	22 (27.2)	39 (48.2)	16 (19.8)	4 (4.9)	Ref		24 (29.6)	29 (35.8)	20 (24.7)	8 (9.9)	Ref		31 (38.3)	40 (49.4)	10 (12.4)	Ref	
Male	26 (21.9)	60 (50.4)	29 (24.4)	4 (3.4)	1.04 (0.54, 2.02)	0.902	48 (40.3)	36 (30.3)	35 (29.4)	0 (0.0)	0.37 (0.19, 0.72)	0.003	46 (38.7)	43 (36.1)	30 (25.2)	1.33 (0.69, 2.56)	0.399
Marital																	
Single	10 (25.0)	20 (50.0)	7 (17.5)	3 (7.5)	Ref		9 (22.5)	9 (22.5)	21 (52.5)	1 (2.5)	Ref		13 (32.5)	17 (42.5)	10 (25.0)	Ref	
Married	38 (23.8)	79 (49.4)	38 (23.8)	5 (3.1)	1.25 (0.59, 2.64)	0.555	63 (39.4)	56 (35.0)	34 (21.3)	7 (4.4)	0.36 (0.17, 0.74)	0.006	64 (40.0)	66 (41.3)	30 (18.8)	0.82 (00.39, 1.70)	0.586
Nursing experience																	
<4 years	25 (20.5)	66 (54.1)	27 (22.1)	4 (3.3)	Ref		46 (37.7)	38 (31.2)	35 (28.7)	3 (2.5)	Ref		49 (40.2)	46 (37.7)	27 (22.1)	Ref	0.000
4–10 years	10 (23.3)	18 (41.9)	11 (25.6)	4 (9.3)	1.43 (0.67, 3.06)	0.351	12 (27.9)	16 (37.2)	12 (27.9)	3 (7.0)	1.89 (0.92, 3.87)	0.083	16 (37.2)	18 (41.9)	9 (20.9)	1.40 (0.66, 2.94)	
>10 years	13 (37.1)	15 (42.9)	7 (20.0)	0 (0.0)	0.76 (0.24, 2.43)	0.640	14 (40.0)	11 (31.4)	8 (22.9)	2 (5.7)	1.61 (0.48, 5.42)	0.443	12 (34.3)	19 (54.3)	4 (11.4)	1.19 (0.38, 3.69)	
Facility																	
Non-covid	16 (31.4)	22 (43.1)	10 (19.6)	3 (5.9)	Ref	0.726	23 (45.1)	14 (27.5)	12 (23.5)	2 (3.9)	Ref	0.018	19 (37.3)	27 (52.9)	5 (9.8)	Ref	0.486
Covid	32 (21.5)	77 (51.7)	35 (23.5)	8 (4.0)	1.15 (0.54, 2.44)		49 (32.9)	51 (34.2)	43 (28.9)	6 (4.0)	2.62 (1.18, 5.83)		58 (38.9)	56 (37.6)	35 (23.5)	1.30 (0.62, 2.71)	

ISI = Insomnia Severity Index; EEQ = Emotional Eater Questionnaire; OSSS-3 = Oslo Social Support Scale 3; OR = Odds ratio; CI = Confidence interval; Ref = Reference category; *p* = *p*-value.

**Table 3:** Adjusted associations between characteristics of nurses and QOL domains, depression, anxiety, and stress scores.

	QOL								Depression		Anxiety		Stress	
	Physical Health β (95% CI)	*p*	Psychological β (95% CI)	*p*	Social Relationships β (95% CI)	*p*	Environment β (95% CI)	*p*	β (95% CI)	*p*	β (95% CI)	*p*	β (95% CI)	*p*
Age														
20–30	Ref		Ref		Ref		Ref		Ref		Ref		Ref	
31–40	−0.5 (−6.0, 4.9)	0.855	0.6 (−5.6, 6.8)	0.848	−3.4 (−10.9, 4.3)	0.386	−1.3 (−7.0, 4.3)	0.663	−0.6 (−2.3, 1.0)	0.427	−0.9 (−2.5, 0.8)	0.292	0.1 (−1.7, 2.0)	0.884
>40	4.9 (−5.9, 15.8)	0.376	10.0 (−3.1, 23.2)	0.146	3.4 (−12.2, 18.9)	0.677	8.6 (−2.4, 19.6)	0.137	−3.2 (−6.7, 0.4)	0.073	−2.8 (−5.9, 0.2)	0.079	−2.8 (−5.7, 0.2)	0.056
Gender														
Female	Ref		Ref		Ref		Ref		Ref		Ref		Ref	
Male	3.1 (−2.1, 8.2)	0.272	−1.3 (−6.2, 3.5)	0.605	−3.4 (−11.4, 4.5)	0.392	−1.6 (−6.6, 3.4)	0.528	−0.2 (−1.8, 1.5)	0.852	−0.9 (−2.5, 0.7)	0.268	−1.5 (−3.2, 0.2)	0.107
Marital status														
Single	Ref		Ref		Ref		Ref		Ref		Ref		Ref	
Married	−0.5 (−6.7, 5.7)	0.871	0.1 (−6.7, 6.8)	0.990	10.8 (3.4, 18.2)	0.007	1.1 (−5.1, 7.3)	0.749	−0.7 (−2.3, 0.9)	0.445	−1.2 (−2.8, 0.5)	0.165	−1.6 (−3.5, 0.3)	0.086
Nursing experience														
<4 years	Ref		Ref		Ref		Ref		Ref		Ref		Ref	
4–10 years	−6.2 (−12.5, 0.1)	0.050	−3.7 (−9.7, 2.2)	0.186	−5.6 (−15.0, 3.8)	0.247	−3.8 (−10.0, 2.4)	0.209	1.8 (−0.2, 3.8)	0.073	0.8 (−0.9, 2.6)	0.379	1.5 (−0.5, 3.5)	0.141
>10 years	−0.2 (−10.7, 10.2)	0.963	−9.6 (−21.7, 2.5)	0.110	−1.3 (−16.5, 13.9)	0.868	−6.8 (−16.8, 3.3)	0.195	2.7 (−0.9, 6.3)	0.134	2.5 (−0.7, 5.7)	0.119	1.5 (−1.0, 4.1)	0.221
Facility														
Non-Covid-19	Ref		Ref		Ref		Ref		Ref		Ref		Ref	
COVID-19	2.0 (−3.9, 7.8)	0.512	6.0 (−1.2, 13.2)	0.098	8.8 (−0.3, 17.9)	0.068	5.4 (−1.5, 12.2)	0.146	−0.2 (−2.5, 2.1)	0.865	1.2 (−0.8, 3.2)	0.241	0.6 (−1.5, 2.7)	0.555

QOL = Quality of life; β = Regression coefficient; CI = Confidence interval; Ref = Reference category; *p* = *p*-value.

## Data Availability

All data generated during this study are included in this published article.
